# Is treatment outcome improved if patients match themselves to treatment options? Study protocol for a randomized controlled trial

**DOI:** 10.1186/s13063-018-2592-9

**Published:** 2018-04-06

**Authors:** Morten Ellegaard Hell, William R. Miller, Bent Nielsen, Anette Søgaard Nielsen

**Affiliations:** 1 Unit of Clinical Alcohol Research (UCAR), Institute of Clinical Research, University of Southern Denmark, Odense, Denmark; 2 Odense Patient data Explorative Network (OPEN), Odense University Hospital, Odense, Denmark; 3 Psychiatric Research Unit, Region of Southern Denmark, Odense, Denmark; 4 Department of Psychology, University of New Mexico, Albuquerque, NM USA

**Keywords:** Alcohol addiction, Binge drinking, Alcohol treatment, Informed choice, Randomized controlled trial

## Abstract

**Background:**

Research on matching patients to treatment has shown that matching grounded in expert views is little better than allocating patients by chance. Furthermore, there is growing emphasis on involving patients in their own treatment as a key to health behavior change. Research on the benefit of having patients choose their treatment from among options, in contrast to being assigned to a treatment by experts, has been limited. Consequently, we designed a rigorous test of patient self-matching to determine whether it does improve retention, adherence, and outcome in alcoholism treatment.

**Methods/design:**

The present study is being conducted as a randomized controlled trial. Four hundred consecutive patients aged 18 years or older will be enrolled and randomized to either self-matching or expert-matching to one of five different treatment approaches. All patients entering the alcohol outpatient clinic in Odense are offered the opportunity to participate in the study. Exclusion criteria are cognitive dysfunction as measured with the Mini Mental State Examination, and non-Danish- or non-English-speaking individuals. The following instruments will be administered at intake to provide standardized measures of alcohol problems: the Addiction Severity Index, Timeline Followback, the World Health Organization quality of life questionnaire, the NEO Five-Factor Inventory 3, and the Personal Happiness Form. For each outcome measure, two analyses will be conducted. Intention-to-treat analyses (ITT) will be carried out with all patients, regardless of whether they complete the interventions or are reinterviewed. Regarding incomplete data, multiple imputations will be used together with ITT analysis. Completer analyses will also be carried out with patients who complete their respective interventions. The primary outcome is decrease in number of monthly excessive drinking days 6 months after initiation of treatment. Secondary outcomes are compliance and 2 quality of life. The influence of personality traits on outcome will also be examined in both groups.

**Discussion:**

The debate on matching patients to treatment has been going on for decades. This study will cast light on this issue by focusing on patients’ choice and thereby clarifying if patients’ perceived autonomy yields better outcomes.

**Trial registration:**

ClinicalTrials.gov, NCT03278821. Registered on 12 September 2017.

**Electronic supplementary material:**

The online version of this article (10.1186/s13063-018-2592-9) contains supplementary material, which is available to authorized users.

## Background

International emphasis is growing on involving patients in their own treatment as a key to health behavior change and improved self-management of chronic diseases [1, 2]. The central concepts are that patients should take an active role in choosing and implementing their own care and that health care systems should actively support and honor patients’ self-determination [3–5].

A related line of research in alcoholism treatment pertains to patient-treatment matching. On the basis of the idea that different kinds of patients may respond differently to different treatment approaches, the U.S. National Institute on Alcohol Abuse and Alcoholism funded the largest randomized trial of alcoholism treatment methods ever conducted: Project MATCH [6]. The primary purpose of this project was to test matching hypotheses regarding which patients would respond optimally to three very different treatments: cognitive behavioral therapy, 12-step facilitation therapy, and motivational enhancement therapy. The principal investigators of this trial, who were prominent alcoholism treatment researchers in the United States, generated two dozen *a priori* matching hypotheses. The project included replication of nearly every matching effect that had been reported in prior clinical trials [7] and represented top experts’ predictions regarding which patients should be assigned to which treatments. The stunning finding of Project MATCH was that even with a very large sample size (*N* = 1726), very few of the hypotheses were confirmed as statistically significant [8, 9]. Nearly all previously reported matching effects failed to be confirmed, and several emerged in a direction opposite to prediction. In a partial replication of the MATCH study, the UK Alcohol Treatment Trial (UKATT) research team conducted a pragmatic randomized trial with two different treatment options [10]. They similarly found improvement in alcohol consumption with all treatment methods, with no difference between the groups or confirmation of matching predictions. In other words, the best “guesses” of some of the field’s most experienced alcoholism treatment researchers in two nations were little better than chance when it came to choosing the best treatment approaches for patients.

Another major attempt at expert matching was a set of patient placement criteria promulgated by the American Society of Addiction Medicine [11]. As it became clear that the outcomes of inpatient programs were no different, on average, from those of less costly outpatient options [12], insurance companies dramatically reduced reimbursement for inpatient treatment. In response, a group of treatment program directors in northern Ohio developed a consensus set of decision rules, known as the *Cleveland criteria* [13], to defend the allocation of patients to particular levels of care. These rules were subsequently published by the American Society of Addiction Medicine [14] and were then revised into even more complex decision systems [15, 16]. Six types of data from an extensive evaluation are used to recommend which of six levels of care a patient should receive.

If an expert matching system such as this one is valid, then patients who are matched to treatment on the basis of these criteria should have better outcomes than those who are mismatched. The few studies of these criteria to date have provided little evidence for their efficacy. One early study [17] demonstrated no significant improvement in 6-month outcomes for matched versus mismatched cases given outpatient treatment (level II, Cleveland criteria). Similarly, only one of several potential matches was statistically significant in another study of inpatient treatment [18]. These studies were limited to evaluation of one level of care. Four trials with patients receiving different levels of care also found little support for this expert matching system. Two of these reported only one “significant” match of the many tested, with no Bonferroni correction for the number of hypotheses tested [19, 20]. The third, a random assignment trial, found no significant matches at all [21]. The fourth, a multicenter observational follow-up study, found that only 24.4% of patients were matched properly to the treatment planned [22]. One naturalistic study in Norway [23] comparing only two levels of care found that cases given less intensive treatment than recommended showed higher attrition, less improvement in severity, and no significant reduction in substance use when compared with matched cases who were offered the recommended level of care. In summary, the findings are quite mixed, and once again, a complex expert system for placing patients into the best treatment for them appears to be little better than chance.

The difficulties experts have in matching patients to treatment and developing useful algorithms have led many researchers to question whether matching based on patient characteristics is optimal for improving treatment outcome [24, 25]. Are there other aspects of treatment and treatment allocation that are more important? If experts are not particularly good at deciding which alcoholism treatment is best for patients, how else might matching be done?

One option is, for instance, to allow patients to match themselves, to make an informed choice from among a menu of evidence-based treatment options. This may not be feasible in smaller programs that may have few staff members; however, in larger systems where more than one treatment option can be provided, it would be feasible to allow patients to choose for themselves. Patient preference is increasingly being considered as good practice in health care, such as when there are several cancer treatment options available with similar overall evidence of efficacy. Patients can be given a fair description of the options open to them and permitted to make an informed choice of which treatment they prefer.

It is still not clear if taking patient preferences into account when choosing between treatment options improves treatment outcome. Authors of a systematic review of the literature on shared decision making in treatment of substance use disorder [26] reported that only 3 of 25 trials revealed a significant effect when treatments were matched to patients’ preferences. The authors stated, however, that the results should be interpreted with caution owing to heterogeneity of the included studies. One study [27] showed that informed choice improved adherence and reduced the amount of smoked cigarettes in a smoking cessation intervention program for patients in treatment for chemical dependency. On the contrary, a study [28] of women with alcohol use disorder, who were given the opportunity of choosing between individual therapy or conjoint treatment with their male partner, showed that the number of patients enrolled in treatment increased, but there was no additional improvement in adherence or reduced drinking days. The two groups also differed significantly in sociodemographic variables that could influence outcome.

There are at least two good reasons for offering patients a choice when the treatment goal is behavior change. The first is that patients are likely to have some wisdom about which behavior change approach is most likely to work for them. Who knows them better? They certainly would know which approaches sound more acceptable or attractive to them. Second, there can be an inherent motivational advantage of choosing one’s own course of action. As Miller et al. stated,

When people perceive that they have freely selected, from among options, a product or course of action, they are likely to be more satisfied with it and committed to it. There is evidence that taking active steps toward change increases the likelihood of successful change, no matter what the action happens to be. If what matters is that the client do *something* and stick with it, then it makes sense to allow clients to select that to which they will be most committed [29].

To our knowledge, however, research is limited on the benefit of having patients freely choose their treatment approach from among options, in contrast to the usual practice of their being assigned to a treatment based on expert clinical judgment. Because there is little evidence that clinical judgment is more effective than chance in choosing optimal treatments, it is reasonable to conduct a rigorous test of patient self-matching to determine whether it does indeed improve retention, adherence, and outcome in alcoholism treatment. As part of the RESCueH studies [30], in the present randomized clinical trial, we will compare the efficacy of patient self-matching versus treatment-as-usual expert matching.

### Purpose and hypotheses

The primary purpose of this randomized controlled trial is to determine if patient self-matching to psychotherapy treatment methods improves drinking outcome, compliance, and quality of life for patients being treated for alcohol problems compared with assignment to treatment as usual, which is by means of expert matching.

#### A priori hypotheses

Our a priori hypotheses are as follows:

1. Patients who choose their own treatment will show significantly greater reductions in alcohol consumption (measured by number of days with excessive drinking) at follow-up, when compared with patients assigned to treatment by expert matching.
2. Patients who choose their own treatment method will show significantly better compliance in treatment (measured by retention) when compared with patients assigned to a specific treatment method by expert matching.

## Methods/design

### Study design

The present study is being conducted as a randomized controlled trial. Four hundred consecutive patients aged 18 years or older will be enrolled. All new patients fulfilling the inclusion criteria will receive oral and written information about the study.

#### Participants

All consecutive patients seeking treatment in the alcohol treatment center in Odense, Denmark, will be offered the opportunity to participate in the study if they meet all of the inclusion criteria below:

1. Fulfill *Diagnostic and Statistical Manual of Mental Disorders, Fourth Edition,* criteria for alcohol abuse or dependence
2. Aged 18 years or older
3. Danish- or English-speaking
4. Agree to participate in the study

The exclusion criteria were as follows:

1. Severe psychosis, measured by clinical interview conducted by psychiatrist
2. Cognitive impairment, measured with the Mini Mental State Examination

#### Setting

Approximately 350 patients start treatment at the alcohol treatment center in Odense every year. Most patients are native Danish speakers. Treatment is provided free of charge, and patients can stay anonymous unless pharmacological treatment is needed. Patients are assigned to one of five treatments according to an algorithm that matches treatment to patient characteristics.

##### Treatment in the outpatient alcohol treatment center

The treatment center offers a range of treatment courses. Patients can be referred for family therapy, cognitive behavioral therapy, contract therapy, supportive therapy, or *netværket* (the network). None of the treatments includes group therapy, and all are provided on an outpatient basis. Whereas family therapy, cognitive behavioral therapy, contract therapy, and supportive therapy are all based on regular sessions with a therapist, *netværket* is a more informal treatment offer. *Netværket* is open 4 days every week, offers the possibility of meeting a therapist but also the possibility of meeting with other patients and carrying out social activities together such as gardening work and other activities. The contact with therapists in *netværket* is relatively brief as compared with the other treatment options. All treatment options involve an interdisciplinary team of nurses, psychiatrists, and social work professionals [31].

After psychiatric evaluation, the patients may also be offered pharmacological treatment, including options of disulfiram, naltrexone, acamprosate, and selective serotonin reuptake inhibitors. This is the same for both the self-match group and the expert match group, as well as for patients not participating in the Self-Match Study.

The duration of treatment and frequency of sessions follow the usual guidelines for outpatient alcohol treatment at the treatment center. Therapists are well educated and trained, and they are experienced in the treatment methods. Frequent supervision to secure therapist fidelity to treatment method takes place. Clinical guidelines are available for all treatment methods.

#### Usual referral routines

On admission, patients may require detoxification treatment or may be ambivalent about seeking treatment at all. After withdrawal symptoms have abated and one or two sessions of motivational interviewing have been completed, patients will decide whether to receive any treatment for their alcohol use disorder.

For those who wish to receive further treatment, the first baseline interview is carried out. Patients will receive oral and written information about the Self-Match Study and then decide whether they want to participate. Patients who agree to participate are randomized to either self-matching or expert matching. All patients, regardless of whether they agree to participate, will have personality traits measured using the NEO Five-Factor Inventory 3 (NEO-FFI-3) [32].

At the second baseline interview, patients’ sociodemographic data, alcohol consumption in the last 30 days, and prior treatment history are clarified. These data are used in the algorithm for expert matching. This interview is also mandatory regardless of whether patients participate in the Self-Match Study because the data are used to plan specific treatment sessions in all the treatment offers. Furthermore, this design ensures that expert matching is treatment as usual. All treatment methods in the study are part of the normal treatment modalities offered at the center, which means that patients who choose not to participate in the study will receive the same treatment as participants. They are assigned to treatment by algorithm as usual.

#### Experimental referral routines

##### Self-matching

At the first baseline interview, the five treatment options that are offered by the treatment center are presented to patients in a short video presentation conducted by the first author. The content of the videos was transcribed and analyzed for readability (LIX number). All five presentations were scored at the same LIX number of 44. The first author or a research assistant will answer any further questions about the format of all treatment options, logistics, and other aspects, but no specific choice of either of the treatment options will be or recommended or favored. The first author or a research assistant will stress that all five treatment methods are evidence-based and effective, at least for some patients. As quality assurance, patients will complete a questionnaire regarding the information they received when asked to make a choice.

#### Study procedures

Information about the study will be presented when the potential participant first attends the treatment center. If the patient needs treatment for withdrawal symptoms, the information will not be given until those symptoms have been sufficiently treated.

**Fig. 1:**
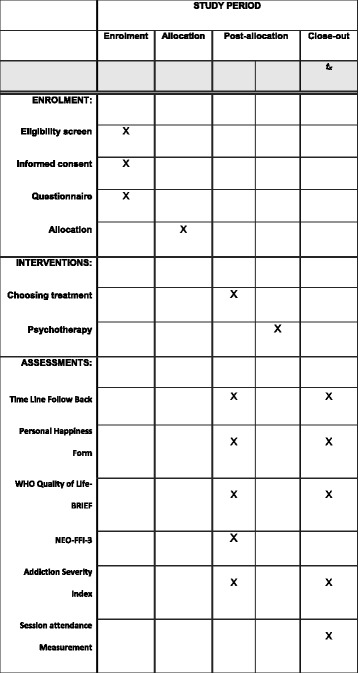
Standard Protocol Items: Recommendations for Interventional Trials: content for the schedule of enrollment, interventions, and assessments. *NEO-FFI-3* NEO Five-Factor Inventory 3, *WHOQOL-BREF* 26-item World Health Organization Quality of Life questionnaire

**Fig. 2:**
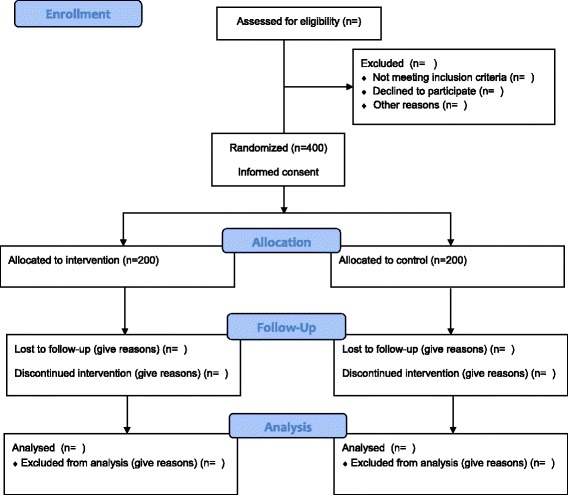
Consolidated Standards of Reporting Trials 2010 flow diagram

The baseline interview is divided into two sessions to avoid fatigue during a long assessment. This is already standard procedure in the clinic for everyone receiving treatment, regardless of participation in the study. Upon providing written and oral consent, patients are randomized to either choosing their own treatment from among five options or being assigned to one of these options by means of the algorithm and expert opinion. Patients participating in the study will be reinterviewed 6 months after initiation of treatment. For an overview, *see* the Standard Protocol Items: Recommendations for Interventional Trials (SPIRIT) in Fig. 1, the flowchart in Fig. 2, and the SPIRIT checklist in Additional file 1.

Getting patients to participate in studies and getting a high follow-up rate is usually an obstacle in research; thus, we have designed the study routines as similarly as possible to the usual assessment routines in the participating treatment institution. The only difference in participation is the 45-minute follow-up interview. To ensure a high follow-up rate, we are collecting phone numbers as well as e-mail and home addresses of patients and their next of kin. If patients decide to remain anonymous, we underline the importance of their responsibility to contact the treatment center 6 months after treatment start.

##### Randomization and blinding

Patients are assigned to conditions by REDCap (Research Electronic Data Capture) from the Odense Patient Data Explorative Network (OPEN), a computer-based randomization system. The interviewer in charge of the first baseline interview will activate the randomization when the patient has agreed to participate in the study. The patient witnesses the randomization, and the result is revealed immediately.

A case report for each participant will be prepared and labeled with the participant’s number. All case reports will be stored in a locked storage facility inaccessible to the therapists, who are to remain uninformed of how patients were assigned to treatment. Patients are urged not to reveal their group assignment.

### Data

The following instruments will be administered to provide standardized measures of alcohol problems, quality of life, and personality traits:

- The Addiction Severity Index (ASI) is an assessment tool for addiction problems [33].
- Timeline Followback is an assessment tool for measuring the number of drinking days during the preceding 30 days [34].
- The 26-item World Health Organization Quality of Life questionnaire [35]
- NEO-FFI-3 is a tool for measuring personality traits [36].
- The Personal Happiness Form is a tool for assessing well-being [37].

These validated and widely used instruments will allow direct comparisons with mainstream clinical trials.

### Statistical analyses and sample size

A multiple regression model will be used to model the percentage of days with drinking/excessive drinking. If the model validation shows that Gaussian multiple regression does not fit owing to severely nonnormal data, a multiple quantile regression model will be used instead. Both modeling approaches allow for inclusion of additional confounders. A backward elimination strategy will be employed to identify significant explanatory variables using a significance level of 0.05. Generally, the two-sided alternative will be considered, except when comparing self-matching with expert matching. In the latter case, a one-sided alternative will be used. Explanatory variables considered will include age, sex, and other relevant available variables.

#### Power calculation

To our knowledge, no similar experimental studies have been conducted. The power calculation is therefore estimated from what clinicians regard to be a clinically meaningful difference in outcome. Miller and Manuel [38] found that clinicians estimated the difference between two treatment methods to be meaningful for implementation in daily practice if the continuous outcome measures (e.g., number of days with excessive drinking) were halved. The power calculation is based on the number of days with excessive alcohol abuse over the last 30 days after 6 months of treatment.

**Fig. 3:**
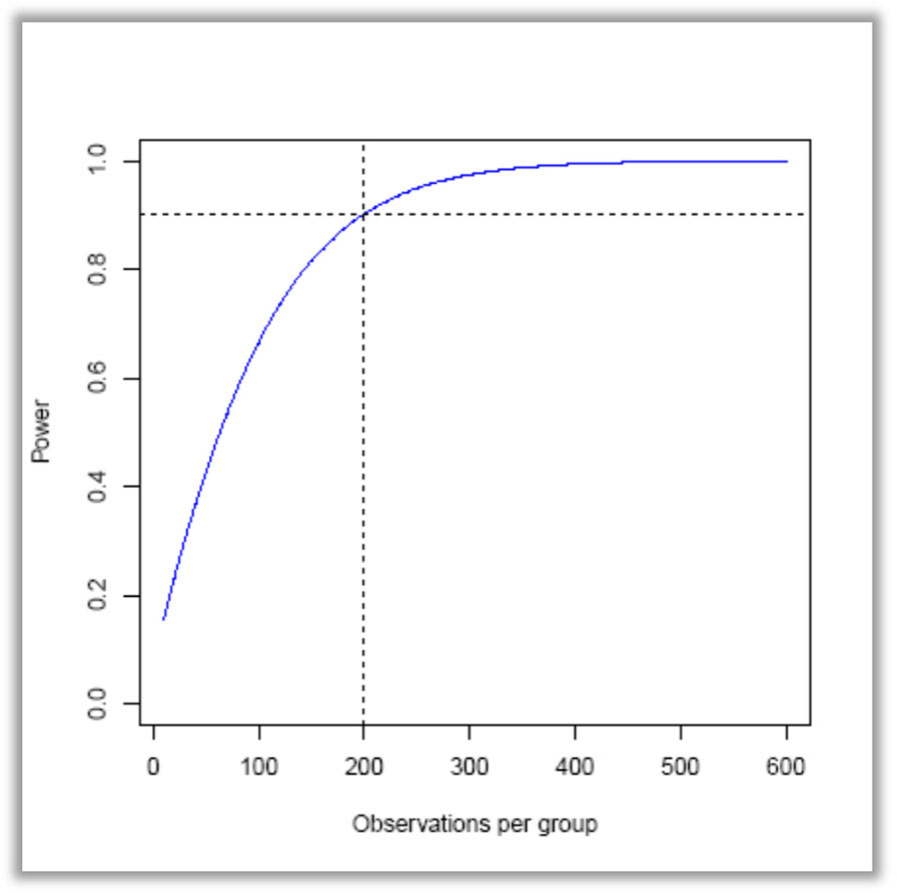
Power calculation

Currently, patients at the participating outpatient clinic drink excessively, on average, 5.7 days (SD 9.7) over the last 30 days after 12 months of treatment. With the new methods for assigning treatment to patients, we seek to halve the number of days with excessive drinking to an average of 5.7/2 = 2.85 days (i.e., a reduction of 2.85 days). We assume the SD will be the same for the reduced number of days. Most likely it will be smaller, because less than zero days of excessive drinking is impossible; that is, the method is conservative. As stated by Miller and Manuel [38], this power calculation is based on practitioners’ judgment of a meaningful difference in outcome rather than on statistical significance based on other studies. By this approach, a total sample of 200 patients in each group is needed to have 90% power to detect a difference of this magnitude using a 0.05% level of statistical significance (Fig. 3). We expect that the proportion of patients assigned to each of the five treatments will differ between the two groups; hence, type of treatment, sociodemographic data, and problem severity will be integrated into the analysis as explanatory variables.

#### Outcome measures

For each outcome measure, two analyses will be carried out:

1. Intention-to-treat analyses (ITT) will be carried out on all patients, regardless of whether they complete the interventions or are reinterviewed. Regarding incomplete data, multiple imputation will be used together with ITT, but there will be some caveats.
2. Completer (on-treatment) analyses will be carried out on patients who complete the respective interventions.

The results will be published, regardless of the findings.

##### Primary outcome

The primary outcome of the study is the number of excessive drinking days 6 months after initiation of treatment.

##### Secondary outcomes

The secondary outcomes of the study are as follows:

1. Compliance as measured by retention: At 6-month follow-up, the number of sessions attended will be measured
2. Quality of life

Deviations in chosen treatment in the self-matching group in relation to anticipated expert matching will be controlled for. In addition, the influence of personality traits on outcome will be explored in both groups.

## Discussion

The debate on matching patients to treatment has been going on for decades. Several studies, including Project MATCH [6] and UKATT [10], have shown that matching patients to treatment is little better than chance, and they have not been able to clarify which aspects of matching improve outcome. In the present study, we will investigate the importance of patient *personal choice* rather than clinician matching patient to treatment based on patient characteristics. Hence, the present study will cast light on whether patients’ perceived autonomy yields better treatment outcome. In addition, personality traits will be measured to investigate whether they influence the impact of patients’ free choice on treatment outcome.

### Challenges

On the basis of a pilot study of 16 patients that showed a higher preference for supportive therapy in the self-matching group than in the expert matching group, we expect that the proportion of patients assigned to the five treatments may differ between the two groups. This will be controlled for in the analysis to clarify whether it is the therapy method or self-matching that causes any observed difference in outcome.

Some would argue that 50% reduction in excessive drinking days is optimistic and therefore would criticize the power calculation. The power calculation is based on clinicians’ estimation of what would constitute a clinically meaningful outcome, however, rather than on statistical significance derived from former studies.

### Innovative aspects

All patients who seek treatment at the center will undergo exactly the same intake procedure, including baseline interviews, for this study. This procedure will provide an opportunity to generate a hypothesis of motives to participate or not participate in studies, because the difference between participating or not is reduced to a single 6-month follow-up interview that will take about 45 minutes to complete. Another innovative aspect is that personality traits will be compared with outcomes in both groups and thereby will provide information on any differences in personality traits of those who profit from self-matching and those who profit from expert matching.

## Trial status

The second version of the protocol was accepted on 17 November 2016. The study was accepted by the Regional Scientific Ethical Committee for Southern Denmark on 24 March 2017. Recruitment began on 22 May 2017 and is expected to end on 30 June 2019. There is currently 196 subjects enrolled and 62 of 69 subjects have participated in the follow up interview.

## Additional file

Additional file 1: SPIRIT 2013 checklist: recommended items to address in a clinical trial protocol and related documents. (DOC 121 kb)

## Abbreviations

ASI: Addiction Severity Index
ITT: Intention-to-treat analyses
NEO-FFI-3: NEO Five-Factor Inventory 3
OPEN: Odense Patient Data Explorative Network
REDCap: Research Electronic Data Capture
SPIRIT: Standard Protocol Items: Recommendations for Interventional Trials
UKATT: UK Alcohol Treatment Trial
WHOQOL-BREF: 26-item World Health Organization Quality of Life questionnaire
